# Effectiveness of short sprint interval training in women with major depressive disorder: a proof-of-concept study

**DOI:** 10.3389/fpsyt.2024.1356559

**Published:** 2024-04-15

**Authors:** Jéssica Alves Ribeiro, Felipe Barreto Schuch, Laís Tonello, Kleber F. Meneghel Vargas, Silvio A. Oliveira-Junior, Paulo T. Müller, Daniel Boullosa

**Affiliations:** ^1^ Graduate Program of Movement Sciences, Integrated Institute of Health, Federal University of Mato Grosso do Sul (UFMS), Campo Grande, Brazil; ^2^ Department of Sports Methods and Techniques, Federal University of Santa Maria, Santa Maria, Brazil; ^3^ Institute of Psychiatry, Federal University of Rio de Janeiro, Rio de Janeiro, Brazil; ^4^ Faculty of Health Sciences, Universidad Autónoma de Chile, Providência, Chile; ^5^ Medicine Department, Universidade de Gurupi - UnirG, Tocantins, Brazil; ^6^ Maria Aparecida Pedrossian Hospital, Federal University of Mato Grosso do Sul, Campo Grande, Brazil; ^7^ Faculty of Physical Activity and Sports Sciences, Universidad de León, León, Spain

**Keywords:** major depressive disorder, high-intensity interval training, physical activity, physical fitness, sprint interval training

## Abstract

**Background:**

High-intensity intermittent training has emerged as an option for treating major depressive disorder (MDD). However, short sprint training (sSIT), an efficient HIIT modality, has not been tested yet for this purpose. The sSIT has been proven to induce the same metabolic adaptations, with the advantage of promoting lower muscle fatigue than other HIIT protocols.

**Methods:**

Seventeen adult women diagnosed with moderate/severe MDD were randomly allocated into a sSIT group (n=9) or a control condition (n=8). The sSIT group completed, over two weeks, six 6-10-min sessions which consisted of 3-12 “all out” sprints of 5 s interspersed with low-intensity recovery of 30-45 s. The week before and after the intervention, both groups were evaluated with the Hamilton Depression Rating Scale of 21-itens (HAM-D21), and for physical fitness and incidental physical activity.

**Results:**

The sSIT group exhibited significant improvements for HAM-D21 scores (24.6±8.2 vs. 16.8±10.1), maximum aerobic power (140±15 vs. 155±15 W), countermovement jump (13.0±3.4 vs. 14.9±3.1 cm), % of body fatness (32.4±4.4 vs. 29.3±3.8%), and 4-days number of steps (13,626±11,309 vs. 16,643±15,371) after the training period when compared to the control group.

**Conclusion:**

Less than 1 hour of a sSIT protocol over two weeks have demonstrated to reduce depressive symptoms, while improving aerobic fitness and body composition, and increasing incidental physical activity in a sample of women diagnosed with MDD.

## 1 Introduction

Depression, the leading cause of disability worldwide is at its maximum values of prevalence after the COVID pandemic, with women typically presenting 3-fold incidence than men (1). Currently, pharmacological interventions and psychotherapy are the first line therapeutic approaches for people with depression (2). However, besides their limited effectiveness (3), other well-known barriers for these dominant therapies in clinical practice are their financial costs, the low adherence (4) and side-effects (5). Meanwhile, an emerging body of evidence is supporting the effectiveness of physical exercise, a low-cost therapy without relevant side-effects which improve the quality of life of patients (6, 7). However, adherence to physical exercise for these patients can be also low (8). Therefore, finding physical exercise interventions that facilitate adherence for these patients is mandatory.

A recent review with meta-analysis and meta-regression (6) has suggested that the magnitude of the effects of physical exercise is not inferior to first-line treatments with moderate-to-high effect sizes on depressive symptoms after analyzing a pool of 41 RCTs. However, while the current evidence supports the effectiveness of either aerobic or resistance training (6), there are limited evidence about the most effective training protocols for patients with depression. In another recent review (9), it has been suggested that high-intensity intermittent training (HIIT) is an appealing physical exercise modality as it presents rapid effects on depressive symptoms but with a reduced training dose thus emerging as an efficient physical exercise treatment for these patients. Among the different HIIT protocols, short Sprint Interval Training (sSIT) may be a more suitable option than other HIIT and SIT protocols for the treatment of depression as it has been demonstrated to induce the same aerobic and anaerobic adaptations (10) but with a lower muscle fatigue (11) thus leading to a more positive affective response (12) because of the very short efforts (≤10 s) and session duration (~10 min). Further, the aerobic and anaerobic adaptations of these protocols can be evident after only 6 sessions in two weeks (13). These loading characteristics would favor the adherence of these patients who are typically sedentary (14). However, to the best of our knowledge, while some studies have confirmed the effectiveness of other SIT protocols with sprinting bouts of longer durations (30 s) in patients with depression (15, 16), no study have verified the effects of a sSIT protocol in this population.

Therefore, the aim of the current study was to verify the effects of a sSIT protocol on depressive symptoms, physical fitness components and incidental physical activity (PA) in a group of adult women diagnosed with Major Depressive Depression (MDD). Based on the current evidence with healthy adults, our hypothesis was that the physical exercise group would present lower depressive symptoms and improved physical fitness components after the short physical exercise intervention.

## 2 Methods

### 2.1 Participants

**Figure 1:**
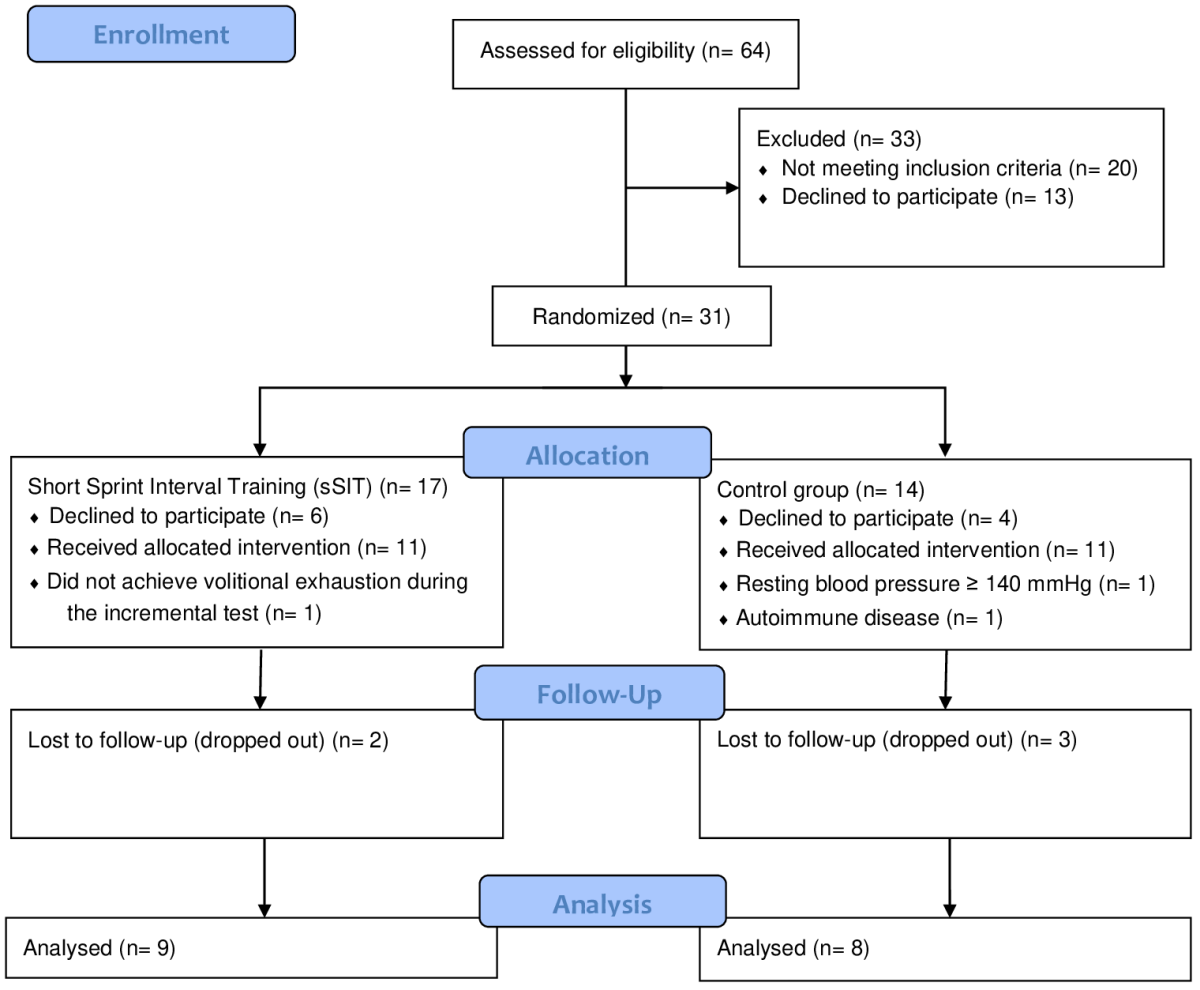
Flow diagram of participants.

Sixty-four women volunteered for participation on this study after seeing advertisements in different media (Instagram, local TV, etc.). Inclusion criteria were being an adult woman with diagnosis of moderate and severe major unipolar depressive disorder (MDD) following the DSM-5 and ICD-10 (F33.1 and F33.2). Exclusion criteria were being pregnant or with symptoms of menopause; be regularly exercising; having a BMI ≥ 35.0 kg/m^2^; presenting a disease or condition that do not allowed participation in the exhaustive test and the physical exercise program or that interfered with the collected variables (e.g. use of a pacemaker; severe stenosis; severe heart failure, etc.); leaving the study at any stage, and not completing the required questionnaires. The patients maintained the use of their medication for the treatment of depression which included a variety of antidepressants and anxiolytics (i.e. Fluoxetine, Bupropion, Sertraline, Desvenlafaxine, Paroxetine, Clonazepam, Alprazolam, Diazepam, and Zolpidem). The Physical Activity Readiness Questionnaire (PAR-Q) was used to identify potential risks and contraindications to perform maximal incremental testing and participate in high-intensity physical exercise (17). Finally, participants were asked about comorbidities and the medication used. After inclusion, they were allocated by simple randomization (i.e. flipping a coin) into an physical exercise or a control group. Seventeen patients concluded the follow-up and were finally included in the control [n=8 (1 month-25 years of treatment)] and the experimental [n=9 (1 month-15 years of treatment)] groups (see **Figure 1**). This study protocol was approved by the Ethics Committee of the Federal University of Mato Grosso do Sul (36637420.5.0000.0021).

### 2.2 Study design

**Figure 2:**
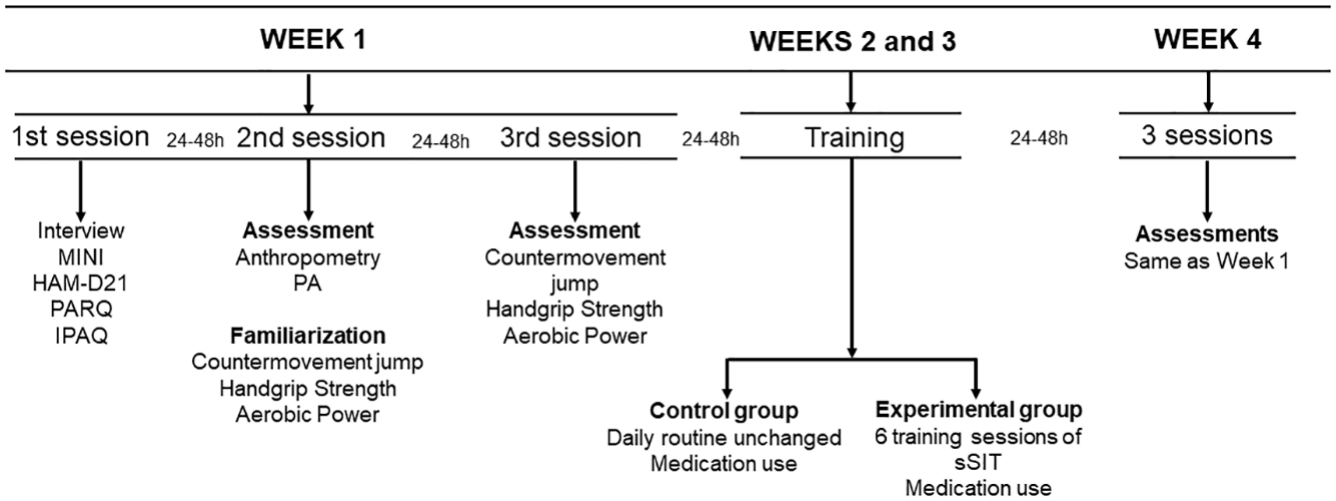
Study design. MINI: International Neuropsychiatric Interview; HAM-D21: 21-item Hamilton Depression Scale; PAR-Q: Physical Activity Readiness Questionnaire; IPAQ: International Physical Activity Questionnaire; PA: Physical activity; sSIT: short sprint interval training group.

The completion of this randomized controlled trial included a 4-week period, with two weeks for clinical and physical evaluations (pre- and post-intervention) and two weeks for application of the experimental protocol (see **Figure 2**). During the application of the experimental protocol, the experimental and control groups were oriented to maintain their habitual lifestyle, including their nutritional habits. During week 1, there were three different evaluation sessions, with an interval of 24-48 hours between them. In the first sessions, questionnaires and interviews were applied by a psychiatrist for inclusion in the study and diagnosis of depressive symptoms. In the second session, participants were familiarized with all the physical fitness tests and were evaluated for body composition. In the third session, they completed all the physical tests. During the second and third weeks, the experimental protocol was applied. Finally, during the fourth week, the evaluations of the first week were repeated. The evaluators were blinded during the participants’ evaluations in the first week, however this was not possible during the fourth week for the physical evaluations only.

### 2.3 Assessments and outcomes

#### 2.3.1 Mental health

A psychiatrist applied the Portuguese version of the International Neuropsychiatric Interview - MINI, for the diagnosis of depression (18). The MINI is a short, structured diagnostic interview developed of choice for psychiatric evaluation and outcome tracking in clinical psychopharmacology trials and epidemiological studies. Permission for its use was formally granted.

The Portuguese version of the 21-item Hamilton Depression Scale (HAM-D21) was the main outcome for depressive symptom assessment (19). The HAM-D21 is a valid and reliable instrument and the most widely used clinician-administered depression assessment scale (20). The scale contains 21 items pertaining to symptoms of depression experienced over the past week to be applied as a structured interview. The cut-off points are: 0-3 normal; 4-7 borderline; 8-15 mild depression; 16-26 moderate depression; ≥27 points: severe depression (20).

#### 2.3.2 Body composition

An anthropometric calibrated scale (LIDER 1050, Brazil) with a precision of 100g and 1cm was used to measure body mass and height. Subsequently, body mass index (BMI) was calculated (kg/m^2^). Waist circumference (WC), abdominal circumference (AC) and hip circumference (HC) were measured with an anthropometric tape (Holtain Ltd., United Kingdom). A calibrated skinfold caliper (Holtain Ltd., United Kingdom) was used for measuring seven skinfolds (pectoral, middle axillary, subscapular, triceps, abdominal, suprailiac and thigh) following the guidelines of the American College of Sports Medicine (21). The % of body fat was subsequently calculated following previously described formulae (22). All these measures were recorded by the same experienced anthropometrist.

#### 2.3.3 Aerobic fitness

The incremental test was performed on a cycle ergometer (INBRAMED – CG4, Brazil) connected to a computer with a custom software. The warm-up started with a load of 0 W for one min, and then the patients pedaled with 20 W of load for two min. After warming up, the patients completed the jump and handgrip evaluations. Subsequently, the incremental test started with 20 W with a progressive increase of 15 W every 2 min, maintaining a fixed cadence of ~60 rpm. During the test, heart rate, systolic and diastolic blood pressures, and rating of perceived exertion (BORG, 1982) were continuously monitored. All the patients were verbally encouraged to physical exercise until voluntary exhaustion (i.e. incapacity to maintain the cadence of ~60 rpm) which was validated with attainment of a maximum heart rate ≥90% of the estimated with a previously validated formula (23). Total time (s) and the maximum aerobic power (W) were recorded for further comparisons.

#### 2.3.4 Countermovement jump

The jump evaluation, a surrogate of lower limbs power, was carried out with the countermovement jump test (CMJ) using the “My Jump2” App (Apple iPhone 12 PRO MAX, USA) which has been validated with the flight time method (24). The patient was asked to jump with maximum effort with the hands placed on their hips. The countermovement depth was freely chosen by the patients (25). The landing was performed with the tiptoes at the same place of take-off. Two jumps were collected with a rest interval ≥15 s and the highest jump was included for analyses (25).

#### 2.3.5 Handgrip strength

Handgrip strength (HGS) was recorded with a calibrated dynamometer (Saehan^®^, Smedley-Type, Korea). The patient was asked to remain in an orthostatic position and to remain immobile throughout the test, without flexing the elbow and shoulder, and without performing shoulder girdle compensation (26). A verbal command was given to the patient who flexed only the finger joints, squeezing the instrument as hard as possible for ~3 s. Three consecutive measurements were collected with a one-minute interval in-between, and the mean value of the three attempts was used for subsequent analyses.

#### 2.3.6 Incidental physical activity

The patients were required to fulfill the short version of the International Physical Activity Questionnaire (IPAQ) to confirm that they were physically inactive. To measure the level of incidental physical activity, a validated pedometer (Digi-Walker^®^ - 700, Yamax, Japan) was used. This pedometer records vertical hip accelerations during gait cycles. The patients were instructed to use the pedometer 3 days a week and one day during the weekend. The placement of the pedometer was standardized by placing it on the waist (27, 28). They were also instructed to remove the pedometer while bathing and sleeping. During the days of recordings, the patients were asked by WhatsApp to not forget these procedures. The total number of steps over the 4 days were included for comparisons between pre- and post-interventions.

### 2.4 Short sprint interval training

The training protocol was completed in the same cycle ergometer of the incremental testing (INBRAMED – CG4, Brazil) and lasted 6-10 min depending on the number of sprints. The warm-up consisted of a 2-min warm-up with 50W of load, at a cadence of ~60 rpm. During each sprint, the patients were instructed to pedal with the maximum possible cadence for 5 s, with strong verbal encouragement by the evaluator. The load imposed during the sprints was the maximum aerobic power (W) achieved during the maximum incremental test. The recovery between sprints lasted 30-45 s and was active with a load of 50W and a cadence of ~60 rpm. At the end of the last sprint, the patients were instructed to spend 2 minutes in active recovery with a load of 50 W.

**Figure 3:**
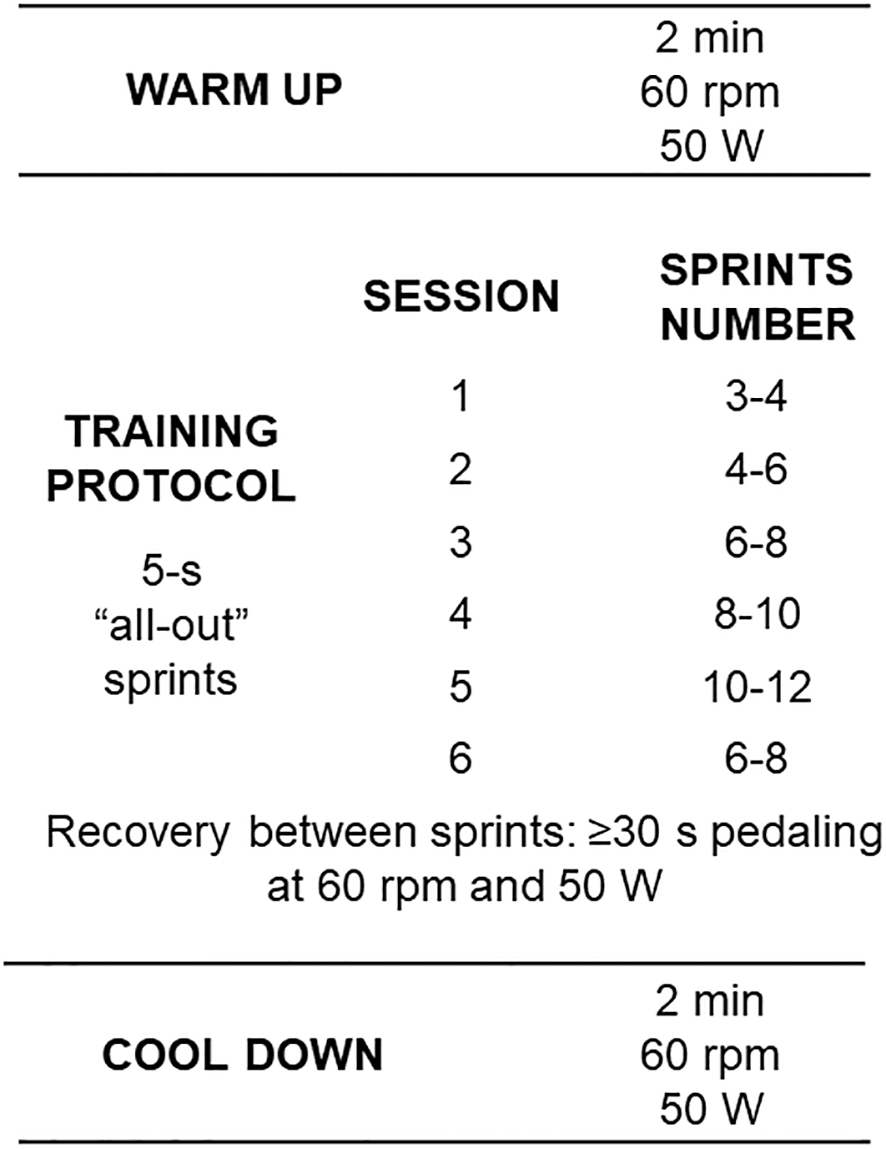
Training protocol with workload characteristics and number of sprints per session. Rpm: revolutions per minute; W: Watts.

The participants performed the sSIT three times a week with a rest of 24 to 48 h between sessions. The training periodization was linear (i.e., the number of sprints were progressively increased with a reduction in the last training session) but individualized as the patients were allowed to select the number of sprints in each session (see **Figure 3**). This protocol was adapted for clinical populations following the original protocol with physically active, young healthy individuals (13). It is expected that only 6 sessions, over two weeks, of this sSIT protocol can induce stable physical (i.e. aerobic and anaerobic performances) and physiological (e.g. VO_2_max, redox status) adaptations (10).

### 2.5 Statistical analyses

Numerical variables were presented as mean and standard deviation (SD), or median and interval between 25th and 75th percentiles, accompanied by 95% confidence intervals (CI 95%). Data normality assumptions were confirmed using the Shapiro-Wilk test (p>0.05). Percentage changes Δ (%) obtained between baseline and post-intervention values were compared using a Student t-test, and a Mann-Whitney test for parametric and non-parametric distributions, respectively. A Two-Way repeated measures (RM) of ANOVA was carried out to evaluate the effects of treatment (sSIT *vs*. control) and moment (week 1 *vs*. week 4). The Bonferroni *post hoc* test was used to make comparisons intra and intergroups. Partial eta squared (*η_p_^2^*) was calculated to determine the effect size (ES). Small, medium, and large effects correspond to η_p_^2^ values of 0.01, 0.06, and 0.14, respectively. All statistical analyses were performed using Sigma Stat for Windows (Version 3.5), and Jamovi^®^ (Version 2.3, available at https://www.jamovi.org). Significance was set at 5% (P < 0.05).

## 3 Results

**Figure 4:**
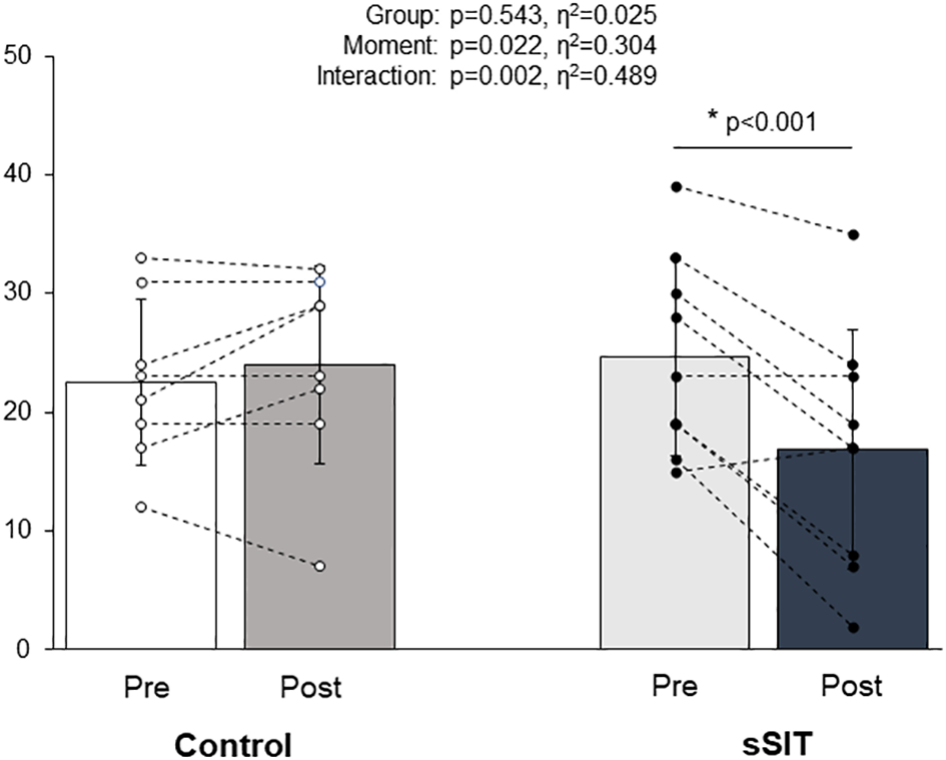
Changes in depression scores (HAM-D21) before (white bars) and after (black bars) the intervention period in the control and experimental groups. HAM-D21: 21-item Hamilton Depression Scale; sSIT: short sprint interval training group; *= p<0.05.

There were no statistically significant differences between groups (Control vs. sSIT) for age (47.0 ± 10.3 vs. 37.1 ± 12.1 yrs; p=0.091), height (161.6 ± 4.2 vs. 163.4 ± 4.8 cm; p=0.422), and body mass (81.0 ± 6.4 vs. 75.7 ± 13.2 kg; p=0.321) before the intervention. The HAM-D21 scores showed a statistically significant reduction in the sSIT group (Pre=24.67 ± 8.29, Post=16.89 ± 10.12; Δ% = -34.30 ± 32.26%) but not in the control group (Pre = 22.50 ± 6.97, Post = 24.00 ± 8.30; Δ% = 5.46 ± 24.66) (see **Figure 4**). There was a significant difference for factor time (F=6.562; p=0.022) and a group × time interaction (F=14.332; p=0.002). Effect sizes for these factors were 0.304 and 0.489, respectively, being classified as large. Pairwise comparisons with the Bonferroni correction revealed that there were pre- to post-changes in the sSIT group (p<0.001) only.

Body composition results are presented in **Table 1**. The sSIT intervention resulted in a lower % of body fatness after the intervention (p<0.05).

**Table 1:** Body composition parameters before and after the treatment in both groups.

Variable	Moment	Group				Factors		
		Control		sSIT		Group	Moment	Interaction
		Mean±SD	CI 95%	Mean±SD	CI 95%			
**Body mass (kg)**	**Baseline**	81.0 ± 6.4	68.4 – 93.6	75.7 ± 13.2	49.8 – 101.7	p=0.295 η_p_^2^=0.073	p=0.284 η_p_^2^=0.076	p=0.534 η_p_^2^=0.026
	**Final**	81.8 ± 7.0	67.9 – 95.6	75.9 ± 12.9	50.7 – 101.2			
	**Δ (%)**	0.89 ± 2.37	-3.8 – 5.5	0.38 ± 2.22	-4.0 – 4.7	p=0.649		
**BMI (kg/m²)**	**Baseline**	31.0 ± 1.4	28.2 – 33.8	28.3 ± 4.5	19.5 – 37.0	p=0.106 η_p_^2^=0.165	p=0.294 η_p_^2^=0.073	p=0.572 η_p_^2^=0.022
	**Final**	31.2 ± 1.4	28.6 – 33.9	28.4 ± 4.4	19.8 – 36.9			
	**Δ (%)**	0.89 ± 2.37	-3.7 – 5.5	0.38 ± 2.22	-4.1 – 4.8	p=0.651		
**WC (cm)**	**Baseline**	87.3 ± 5.6	76.3 – 98.2	81.3 ± 10.1	61.5 – 101.2	p=0.088 η_p_^2^=0.181	p=0.463 η_p_^2^=0.037	p=0.109 η_p_^2^=0.162
	**Final**	88.7 ± 4.6	79.6 – 97.7	80.8 ± 9.2	62.7 – 98.8			
	**Δ (%)	2.34 ± 2.18	-3.5 – 7.0	0.00 ± 2.62	-6.4 – 5.4	p=0.228		
**AC (cm)**	**Baseline**	94.1 ± 6.6	81.2 – 107.0	89.6 ± 11.7	66.7 – 112.5	p=0.175 η_p_^2^=0.119	p=0.464 η_p_^2^=0.036	p=0.104 η_p_^2^=0.167
	**Final**	96.4 ± 6.0	84.7 – 108.1	88.7 ± 10.1	68.8 – 108.6			
	**Δ (%)**	2.50 ± 3.87	-5.1 – 10.1	-0.73 ± 4.49	-9.5 – 8.1	p=0.136		
**HC (cm)**	**Baseline**	108.3 ± 6.8	95.0 – 121.5	104.2 ± 8.5	87.6 – 120.8	p=0.183 η_p_^2^=0.115	p=0.164 η_p_^2^=0.125	p=0.048 η_p_^2^=0.236
	**Final**	110.0 ± 6.7 *	96.8 – 123.2	103.9 ± 7.8	88.6 – 119.2			
	**Δ (%)**	1.63 ± 1.48	-1.3 – 4.5	-0.25 ± 2.17	-4.5 – 4.0	p=0.057		
**Body fatness (%)**	**Baseline**	34.7 ± 1.8	31.2 – 38.1	32.4 ± 4.5	23.7 – 41.2	p=0.025 η_p_^2^=0.293	p=0.068 η_p_^2^=0.205	p<0.001 η_p_^2^=0.558
	**Final**	35.6 ± 1.9	31.9 – 39.4	29.9 ± 4.1 *#	21.9 – 38.0			
	**Δ (%)**	2.80 ± 5.52	-8.0 – 13.6	-7.52 ± 3.87 #	-15.1 – 0.1	p<0.001		

Values are expressed as mean ± SD, and 95% confidence intervals (CI 95%). BMI, body mass index; WC, waist circumference; AC, abdominal circumference; HC, hip circumference; sSIT, short sprint interval training. Two-Way repeated measures of ANOVA and Bonferroni’s test; * p<0.05 vs. Baseline (effect of moment within group); # p<0.05 vs. Control group (effect of group within moment); η_p_^2^, partial eta square. Δ (%) values obtained from the difference between baseline and post-intervention, and compared with Student t test; # p<0.05 vs. Control group. **Δ (%) WC values are expressed as median ± interval between 25th and 75th percentiles and analyzed with a Mann-Whitney test.

Physical fitness and PA measures are presented in **Table 2**. The sSIT intervention resulted in an increased aerobic fitness (i.e. Maximum aerobic power and total time) and incidental PA levels after the intervention (p<0.05). Of note, the CMJ showed a statistically significant change after the sSIT intervention (p<0.05).

**Table 2:** Physical fitness and physical activity parameters before and after the treatment in both groups.

Variable	Moment	Group				Factors		
		Control		sSIT		Group	Moment	Interaction
		Mean±SD	CI 95%	Mean±SD	CI 95%			
**CMJ (cm)**	**Baseline**	10.2 ± 3.0	4.3 – 16.2	13.0 ± 3.4	6.3 – 19.6	p=0.036 η_p_^2^=0.260	p=0.003 η_p_^2^=0.446	p=0.075 η_p_^2^=0.196
	**Final**	10.8 ± 3.1	4.8 – 16.8	14.9 ± 3.1 *#	8.9 – 21.0			
	**Δ (%)**	6.35 ± 12.14	-17.4 – 30.1	17.64 ± 16.03	-13.8 – 49.1	p=0.126		
**HGS (kgf)**	**Baseline**	27.0 ± 3.4	20.3 – 33.7	26.5 ± 4.5	17.0 – 35.4	p=0.828 η_p_^2^=0.003	p=0.813 η_p_^2^=0.004	p=0.093 η_p_^2^=0.176
	**Final**	25.9 ± 4.0	18.0 – 33.8	27.4 ± 6.0	15.6 – 39.1			
	**Δ (%)**	-3.91 ± 9.27	-22.1 – 14.3	2.32 ± 7.97	-13.3 – 17.9	p=0.157		
**AP (W)**	**Baseline**	119 ± 16	88 – 151	141 ± 19 #	103 – 179	p=0.003 η_p_^2^=0.447	p=0.055 η_p_^2^=0.224	p=0.016 η_p_^2^=0.327
	**Final**	118 ± 16	86 – 149	155 ± 21 *#	113 – 197			
	** Δ (%)	0.00 ± 0.00	-9.82 – 6.82	9.68 ± 17.38 #	-15.21 – 37.17	p=0.015		
**Total time (s)**	**Baseline**	514 ± 51	415 – 612	569 ± 75	422 – 716	p=0.008 η_p_^2^=0.382	p=0.004 η_p_^2^=0.427	P<0.001 η_p_^2^=0.526
	**Final**	505 ± 46	415 – 595	653 ± 101*#	456 – 850			
	**Δ (%)**	-1.52 ± 3.52	-8.43 – 5.38	14.94 ± 11.42 #	-7.44 – 37.32	p=0.001		
**Steps**	**Baseline**	13,343±9,511	-5,299 – 31,984	18,843±8,781	1,632 – 36,053	p=0.038 η_p_^2^=0.258	p=0.484 η_p_^2^=0.033	p=0.009 η_p_^2^=0.371
	**Final**	10,181±6,905	-3,352 – 23,715	24,017±10,882*#	2,687 – 45,346			
	** Δ (%)	-14.1 ± 62.5	-243.7 – 303.6	26.7 ± 61.0	-66.8 – 134.4	p=0.136		

Values are expressed as mean ± SD, and 95% confidence interval (CI 95%). CMJ, countermovement jump; HGS, handgrip strength; AP, aerobic power; sSIT, short sprint interval training. Two-Way repeated measures of ANOVA and Bonferroni’s test; * p<0.05 vs. Baseline (effect of moment within group); # p<0.05 vs. Control group (effect of group within moment); η_p_^2^, partial eta square. Δ (%) values obtained from the difference between basal and post-treatment values, and analyzed with a Student t test; **Δ (%) AP, and Steps values are expressed as median ± interval between 25th and 75th percentiles, and analyzed with a Mann-Whitney test; # p<0.05 vs. Control group.

## 4 Discussion

This is the first study exhibiting the positive effects of a sSIT program on symptoms of depression, physical fitness and PA in patients diagnosed with MDD. This study extends the previous evidence demonstrating the improvements in depressive symptoms after other HIIT protocols in people with mental illness (9). However, the current protocol has the advantage of being shorter than other HIIT protocols with only six 6-10-min sessions totalizing less than 1 hour of physical exercise over two weeks. Therefore, the results of the current study are very promising for the treatment of people with depression due to their effects on mental and physical health outcomes combined with a low time demand. This is important as one of the barriers to engaging in an physical exercise program is the lack of time.

Previous studies with other HIIT protocols have demonstrated a similar effectiveness to improve both depressive symptoms and aerobic fitness (9). The current evidence suggests that the neurobiological mechanisms relating depression and physical fitness are meditated by the adaptations of the aerobic metabolism as there is often a weak-to-moderate correlation between maximum oxygen consumption (VO_2_max) and depressive symptoms across different studies (29, 30). Unfortunately, we were unable to evaluate VO_2_max at follow up because of technical issues with the metabolic cart, and therefore cannot explore further whether changes on VO_2_max are associated with changes on depressive symptoms. However, both maximum aerobic power (W) and total time (s) in the incremental test are well-recognized markers of aerobic fitness which are highly correlated to VO_2_max. Furthermore, maximum aerobic power always presents a greater reliability than VO_2_max itself because of the typically high technical error associated to metabolic measurements (31). Accordingly, the improvements in these working capacity parameters exhibited by our participants may be equivalent to ~1 MET following previous estimates (32). While we did not evaluate any metabolic parameter, the improvements in aerobic power experienced by our participants would be more related to peripheral adaptations associated to VO_2_max than central adaptations which are more likely to occur with other HIIT protocols with longer physical exercise bouts (33). Therefore, further studies should explore the associations among metabolic and cardiovascular adaptations after HIIT and SIT protocols with changes of symptoms of depression. Meanwhile, clinicians and other health professionals should be aware of the possibility of evaluating aerobic fitness after physical exercise treatments with working capacity measures during direct and indirect protocols without the need for recording actual VO_2_max values.

One alternative mechanism behind the antidepressive effects of the SIT protocols may be related to lactate. Recently, it has been suggested that lactate, a metabolite derived from glycolysis during anerobic efforts, can promote antidepressive-like effects and enhance resilience to stress via different signaling mechanisms after its administration in animal models (34, 35). While our sSIT protocol has been designed to limit the glycolytic activation during sprinting bouts therefore reducing the associated metabolic fatigue and thus increasing the affective responses (10), it was expected that lactate levels were slightly elevated thus maybe promoting the positive effects observed in animal models. Future studies may test this hypothesis in humans while examining the increase of other hormonal mediators as Brain-Derived Neurotrophic Factor (BDNF) after SIT protocols differing in lactate levels (36, 37) and muscle contraction regimens (e.g. eccentric *vs*. concentric) (38).

Interestingly, the participants from the sSIT group slightly reduced their body fatness while importantly incrementing their incidental PA levels as recorded with their 4-days pedometer recordings. These results of a reduced adiposity after short-term HIIT interventions are not novel since a previous study (39) found a small reduction in body fatness after a Wingate-based SIT protocol of 30-s sprints of only 6 training sessions in a sample of young healthy adults. In addition, another recent study in patients with MDD (16) reported an increased, self-reported PA after a 4-week Wingate-based SIT intervention of 3 training sessions per week. Therefore, while these changes may be expected after SIT interventions, our protocol was more effective since the patients underwent less than 1 hour of physical exercise over two weeks. In this regard, it would be speculated that the changes in body composition would be related, not only to the physical exercise protocol, but also to the increase in PA levels following the physical exercise intervention. Previously, it has been suggested that post-exercise energy expenditure is not compromised after sSIT protocols when compared with protocols of longer sprinting bouts, but a reduced fat oxidation was reported (40). Therefore, it may be speculated that the increased PA levels, as a consequence of the improved mood in these patients, may be the main mechanism behind the reduced body fatness. Meanwhile, we cannot ignore the possibility that the patients changed their nutritional habits and energy intake despite being advised to maintain them during all the intervention. Future studies are warranted to better understand these physical adaptations and their related biological mechanisms.

Interestingly, CMJ, a simple and valid test for neuromuscular performance evaluation of the lower limbs’ power, exhibited a significant change after the intervention. This is not somewhat surprising as a cross-training effect (41) may be expected as frequently exhibited by athletes training different physical exercise modalities. Further, this cross-training effect would be expected to be more evident in clinical populations with very low physical fitness levels as in the current study because of the strength required during sprinting bouts for the lower-limbs. Thus, it may be suggested that, despite using a non-specific method for strength development, the anaerobic development after the sprinting bouts (13) may be potentially promoting strength-related adaptations which would likely become larger after a longer training period. This aspect is valid for this, and other clinical settings using diverse physical exercise interventions and should be considered in further studies.

This study is not without limitations. First, we did not control the energy intake of participants therefore future studies are required to explore the relative influence of physical exercise, PA and nutritional intakes in the changes associated with sSIT protocols. Second, we only included unipolar MDD patients therefore other psychiatric patients with symptoms of depression should be included in future studies. Third, we only recorded maximum aerobic power and total time in the incremental test as indices of aerobic fitness, thus future studies should report VO_2_max, its specific central and peripheral adaptations, and lactate levels to better understand their link with symptoms of depression. Finally, this study presents a limited sample size and time of intervention. Therefore, future studies with greater sample sizes and longer interventions should confirm these important findings. However, it should be pointed out that most patients decided to regularly practice other forms of physical exercise after our sSIT intervention. Meanwhile, the effect sizes reported, the CIs, and their p-values are indicative of robust adaptations in accordance with previous studies using similar sSIT protocols with healthy young individuals (13, 42).

## 5 Conclusions

This study is the first to report significant changes in symptoms of depression after a sSIT protocol of only six 6-10-min sessions over two weeks in female patients diagnosed with MDD. These positive adaptations were associated to changes in aerobic fitness, body fatness and incidental PA levels. Future studies with greater sample sizes and follow-up should confirm the high effectiveness and efficiency of this protocol in this and other psychiatric populations dealing with symptoms of depression.

## Data availability statement

The raw data supporting the conclusions of this article will be made available by the authors, without undue reservation.

## Ethics statement

The studies involving humans were approved by Ethics Committee of the Federal University of Mato Grosso do Sul (36637420.5.0000.0021). The studies were conducted in accordance with the local legislation and institutional requirements. The participants provided their written informed consent to participate in this study. Written informed consent was obtained from the individual(s) for the publication of any potentially identifiable images or data included in this article.

## Author contributions

JR: Writing – review & editing, Writing – original draft, Investigation, Data curation. FS: Writing – review & editing, Writing – original draft, Validation, Methodology. LT: Writing – review & editing, Writing – original draft, Methodology. KM: Writing – review & editing, Writing – original draft, Validation, Supervision. SO: Writing – review & editing, Writing – original draft, Formal analysis, Data curation. PM: Writing – review & editing, Writing – original draft, Supervision, Investigation. DB: Writing – review & editing, Writing – original draft, Supervision, Project administration, Methodology, Funding acquisition, Conceptualization.
